# Phase I investigator-initiated study of the safety of MTC001 in patients with chronic ischemic heart failure

**DOI:** 10.1097/MD.0000000000028372

**Published:** 2021-12-23

**Authors:** Takeshi Machino, Akira Sato, Nobuyuki Murakoshi, Masaki Ieda

**Affiliations:** a Department of Cardiology, Faculty of Medicine, University of Tsukuba, Tsukuba, Japan; b Department of Clinical Research and Regional Innovation, Faculty of Medicine, University of Tsukuba, Tsukuba, Japan; c Department of Cardiology, University of Yamanashi, Yamanashi, Japan.

**Keywords:** autologous cell therapy, cardiac fibroblast, electro-anatomical mapping, first-inhuman trial, heart failure, transendocardial injection, VCAM1

## Abstract

**Background:**

: Heart failure (HF) is a global pandemic most commonly caused by coronary artery disease. Despite coronary revascularization, the infarcted myocardium can develop into an irreversible scar toward chronic ischemic HF. This is due to the limited regenerative capacity of the adult human heart. Recently, the vascular cell adhesion molecule 1 positive cardiac fibroblast (VCF) has been shown to directly improve cardiac contractility in addition to promoting myocardial growth in preclinical studies. This clinical trial aims to explore the safety and, in part, the efficacy of autologous VCF therapy for chronic ischemic HF.

**Methods:**

: This first-in-human trial is an open-label, single-arm, phase 1 study conducted at a single center. This study will include 6 patients with chronic ischemic HF in stage C and NYHA class II or III despite receiving the standard of care, including coronary revascularization. Participants will undergo cardiac biopsy to manufacture autologous VCFs expressing CD90 and CD106. Under electro-anatomical mapping guidance, participants will receive a transendocardial injection of VCF in a modified 3 + 3 design. The first 3 patients will receive a standard dose (2 × 10^7^ cells) of VCF with a 4-week interval for safety assessment before subsequent enrollment. In the absence of safety issues, the final 3 patients will receive the standard dose of VCF without a 4-week interval. In the presence of safety issues, the final 3 patients will receive a reduced dose (1.5 × 10^7^ cells) of VCF with the 4-week interval.

**Discussion:**

This is the first clinical study of cardiac regeneration using VCFs for the treatment of chronic ischemic HF. The study results will contribute to the development of a minimally invasive cell therapy for patients with HF failed by the standard of care.

**Trial registration:**

This study was registered with the Japan Registry of Clinical Trials (jRCT2033210078).

## 1 Introduction

Heart failure (HF) is a global pandemic affecting an increasing number of patients, comprising at least 26 million worldwide.^[1,2]^ The chronic progressive nature of HF exhibits a poor prognosis, with an in-hospital mortality rate of 8% and 1-year mortality rate of 7.3%.^[3,4]^ Furthermore, HF is a leading cause of hospitalization, with a high readmission rate of 27% within 6 months and 35% within 1 year.^[5,6]^ The most common cause of HF is coronary artery disease.^[7,8]^ Infarcted myocardium, despite coronary revascularization, develops into an irreversible scar leading to systolic dysfunction and adverse remodeling toward chronic ischemic HF. This is predominantly due to the limited regenerative capacity of the adult human heart.^[9]^

Recent advances in cardiac regeneration have implicated cardiac fibroblasts (CFs).^[10,11]^ In particular, a CF expressing vascular cell adhesion molecule 1 (VCAM1) has been shown to promote myocardial growth through VCAM1/very late activation antigen 4 signaling and the Janus kinase/signal transducer and transcription pathway activator.^[12]^ This VCAM1-positive CF (VCF) has been reported to promote lymphangiogenesis in infarct lesions.^[13]^ This directly improves cardiac contractility by regulating the fluid homeostasis of the heart.^[14–16]^ Moreover, VCF has been reported to improve systolic function in animal models of ischemic HF via direct injection into the myocardium.^[12,13]^

The present first-in-human trial aims to explore the safety and, in part, the efficacy of a transendocardial injection of autologous VCF in patients with chronic ischemic HF. Herein, we provide a detailed design of this phase 1 clinical trial.

## 2 Methods

### 2.1 Study design

**Figure 1:**
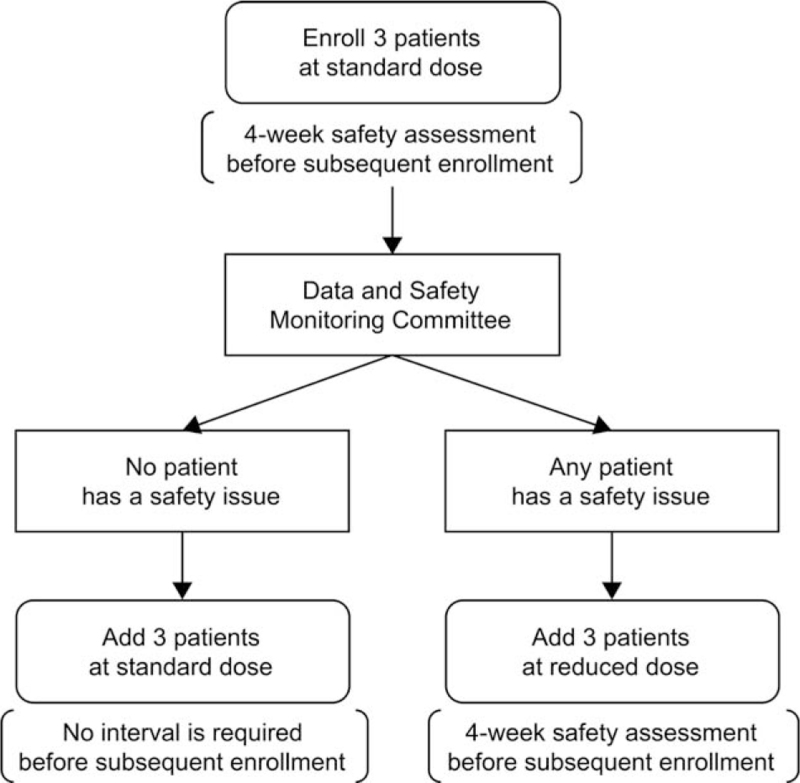
Study design. Each patient will receive a transendocardial injection of vascular cell adhesion molecule-1 positive cardiac fibroblasts at the standard dose (2 × 10^7^ cells) or reduced dose (1.5 x 10^7^ cells) according to the instructions of the Data and Safety Monitoring Committee.

This phase 1 study is an open-label, single-arm, first-in-human trial conducted at a single center (University of Tsukuba Hospital). The safety and, in part, the efficacy of transendocardial injection of VCF will be evaluated at 12 and 52 weeks after the VCF injection. A total of 6 patients with chronic ischemic HF will receive the investigational therapy in a modified 3 + 3 design. The first 3 patients receive a standard dose (2 × 10^7^ cells) of VCF injection, with a 4-week interval for safety assessment set between each patient enrollment. If there are no safety issues in any of the first 3 patients, the final 3 patients receive the standard dose of VCF injection without a 4-week interval between enrollments. On the contrary, if any of the first 3 patients experience a safety issue, the final 3 patients receive a reduced dose (1.5 × 10^7^ cells) of VCF injection, with a 4-week interval for safety assessment between enrollments. The Data and Safety Monitoring Committee independently assessed the safety of investigational therapy to allow for subsequent enrollments and provided standardized dose instructions accordingly (Fig. 1).

### 2.2 Study population

The investigational therapy is assumed to improve the systolic dysfunction, retarding the progression of ischemic HF to the advanced stage (Stage D).^[6,17]^ Therefore, this study includes patients with stage C ischemic HF with reduced systolic function despite coronary revascularization and standard medical therapy. They must be ineligible for or nonresponsive to cardiac resynchronization therapy (CRT). Patients with asymptomatic HF would hardly benefit from the investigational treatment, while patients with severe HF may have a high risk for this safety trial. Therefore, patients with New York Heart Association functional classes II and III are eligible for this study. The eligibility criteria are summarized in Table 1.

**Table 1:** Patient eligibility.

Inclusion criteria
(1) Aged between 20 and 84 years (inclusive) at the time of informed consent
(2) Heart failure with old myocardial infarction
(3) Heart failure in stage C and NYHA functional class II or III
(4) LVEF ≤40% on echocardiography
(5) Ineligible for CRT or nonresponsive to CRT (LVEF improvement <5%)
(6) No need for further coronary revascularization
(7) Treated with standard pharmacotherapy for 3 months or longer
Exclusion criteria
(1) Poorly controlled diabetes (HbA1c >8.5%)
(2) Congenital enzyme abnormality or muscle disease
(3) Active autoimmune disease
(4) Active malignancy
(5) Are or may be pregnant
(6) Wish to become pregnant within 52 weeks after the investigational treatment
(7) Estimated glomerular filtration rate <30 mL/min/1.73 m^2^, or serum creatinine ≥3.0 mg/dL
(8) Hematocrit < 25%
(9) Positive for hepatitis B antigen or detectable HBV-DNA on real-time PCR before the cardiac biopsy
(10) Positive for HCV, HIV, HTLV-1, or syphilis antibodies before the cardiac biopsy
(11) Treated with other gene or cell therapies, etc, for severe HF within the past 2 years
(12) Unable to complete the 6-min walk test due to reasons other than HF
(13) History of serious allergies (contrast media allergy, status asthmaticus, anaphylactic shock, etc)
(14) History of hypersensitivity to antibiotics (penicillin, streptomycin, amphotericin B)
(15) History of hypersensitivity to animal-derived ingredients such as fetal bovine serum
(16) History of hypersensitivity to mouse-derived components
(17) ICD not implanted despite class I indication
(18) Appropriate ICD shock within the past 3 months
(19) Acute coronary syndrome within the past 3 months

CRT = cardiac resynchronization therapy, DNA = deoxyribonucleic acid, HbA1c = hemoglobin A1c, HBV = hepatitis B virus, HCV = hepatitis C virus, HF = heart failure, HIV = human immunodeficiency virus, HTLV-1 = human T-lymphotropic virus type 1, ICD = implantable cardioverter defibrillator, LVEF = left ventricular ejection fraction, NYHA = New York Heart Association, PCR = polymerase chain reaction.

### 2.3 Study intervention

The investigational product, “MTC001,” contains autologous VCF expressing markers of both fibroblast (CD90) and VCAM1 (CD106). Autologous VCF is derived from the patient's endomyocardial biopsy. Up to 4 biopsy samples are obtained from the right ventricular endomyocardial septum via internal jugular vein access using 5.5-Fr endomyocardial biopsy forceps (Cordis Corp., CA). MTC001 is manufactured from cardiac biopsy samples by Metcela Inc. (Kawasaki, Japan). Briefly, primary CFs are enzymatically isolated, expanded, and cultured as previously reported.^[13]^ After the initial expansion, the CFs expressing both CD90 and CD106 are isolated by magnetic-activated cell sorting.

The VCF product (MTC001) is injected into the myocardium around the infarcted scar of the left ventricle via a transendocardial approach with retrograde femoral arterial access.^[18,19]^ Electro-anatomical mapping (EAM) is performed with an EnSite system (Abbott, IL) using a novel bidirectional multipolar 7-Fr mapping catheter (Japan Lifeline Co., Ltd., Tokyo, Japan). An injection needle catheter (Japan Lifeline Co., Ltd., Tokyo, Japan) is advanced through the inner lumen of the mapping catheter toward the scar border zone (0.5–1.5 mV) detected by bipolar EAM.^[20–22]^ The VCF is injected into the myocardium from the side holes around the distal tip of the injection needle catheter. The injection sites are indicated by tags on EAM.

### 2.4 Outcome measurements

The primary endpoint for safety is the incidence of treatment-emergent serious adverse events (SAEs). The composite outcome comprises death, myocardial infarction, stroke, hospitalization for worsening HF, cardiac perforation, pericardial tamponade, sustained ventricular tachycardia (>30 seconds or hemodynamic collapse), and ventricular fibrillation.^[23,24]^ Secondary endpoints for efficacy include left ventricular ejection fraction on magnetic resonance imaging (MRI) or computed tomography (when MRI is not available) as a mechanical assessment, 6-minute walk test and cardiopulmonary exercise testing as functional evaluations, brain natriuretic peptide and N-terminal pro-brain natriuretic peptide as biomarkers of HF, and the Minnesota Living with Heart Failure Questionnaire as a quality of life assessment. Patients are hospitalized for 1 week, with follow-up visits at 4, 8, 12, 24, 36, and 52 weeks after the investigational therapy (Table 2).

**Table 2:**
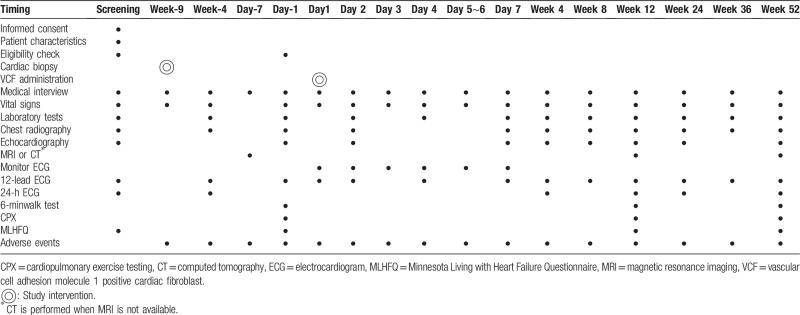
Study schedule.

### 2.5 Adverse events

Adverse events (AEs) are recorded during the study period, from the cardiac biopsy to the final follow-up visit at 52 weeks after VCF injection. AEs are defined as all undesirable diseases, symptoms, and signs, including laboratory tests, regardless of the causal relationship with this study. Worsening of underlying comorbidities and associated reactions are excluded from the AEs. SAEs are defined as death, life-threatening condition, prolonged or additional hospitalization, disability including its risk, and congenital disease or anomaly.

### 2.6 Quality management

A case report form is used to record the data required by the study protocol for each participant. The data sources include medical records, slips, worksheets, consent forms, and comments in the case report form. Data quality is checked by the data manager. The data are finalized after appropriate correction by investigators. The principal investigator has appointed a monitor and an auditor to monitor and audit this clinical trial, respectively.

### 2.7 Ethics and trial status

This study complies with the Declaration of Helsinki, Pharmaceutical and Medical Devices Act, Ministerial Ordinance on Good Clinical Practice (MHW Ordinance No. 28; March 27, 1997), and Ministerial Ordinance on Good Clinical Practice for Regenerative Medical Products (MHLW Ordinance No. 89; July 30, 2014). The study protocol was approved by the University of Tsukuba Hospital Institutional Review Board and registered with the Japan Registry of Clinical Trials (jRCT2033210078). The first version of this protocol was published on May 10, 2021, and was last updated on October 7, 2021. Patient recruitment began in June 2021.

## 3 Discussion

The progressive nature of HF resists the standard of care, which predominantly consists of pharmaceutical approaches, including renin-angiotensin-aldosterone system inhibitors, beta blockers, and diuretics.^[6,17,25]^ Although CRT is an effective treatment option for drug-resistant HF, less than one-third of patients meet the indication criteria. Furthermore, 30% to 40% of patients do not respond to CRT.^[6,26,27]^ The last resort for advanced HF is heart transplantation, which suffers from a chronic shortage of donor hearts and limited eligibility.^[28–30]^ This is rarely complemented by a left ventricular assist device, which is an expensive and invasive option, with a risk of major complications (stroke, bleeding, infection, etc).^[31]^ These issues have facilitated various cardiac regeneration strategies.^[32–34]^ However, none of the proposed strategies have been widely implemented in clinical practice owing to their limited efficacy.^[35]^

VCF can directly improve systolic dysfunction, in addition to promoting myocardial growth.^[12–16]^ Indeed, myocardial injection of VCF significantly improves systolic dysfunction in ischemic HF models.^[12,13]^ Accordingly, this first-in-human trial was designed to explore the safety and, in part, the efficacy of a transendocardial injection of VCF in patients with chronic ischemic HF. Transendocardial injection guided by EAM serves as a minimally invasive method for precise administration. We hope that the present study provides a basis for further clinical trials of this strategy to treat patients with ischemic HF failed by the standard of care.

## Acknowledgments

The authors would like to thank the staff at the Tsukuba Clinical Research and Development Organization for their support.

## Author contributions

**Conceptualization:** Takeshi Machino, Akira Sato, Nobuyuki Murakoshi.

**Funding acquisition:** Takeshi Machino, Akira Sato.

**Investigation:** Takeshi Machino, Nobuyuki Murakoshi.

**Methodology:** Takeshi Machino, Nobuyuki Murakoshi.

**Project administration:** Takeshi Machino.

**Supervision:** Akira Sato, Masaki Ieda.

**Writing – original draft:** Takeshi Machino.
